# Compassionate use of JAK1/2 inhibitor ruxolitinib for severe COVID-19: a prospective observational study

**DOI:** 10.1038/s41375-020-01018-y

**Published:** 2020-08-19

**Authors:** Alessandro M. Vannucchi, Benedetta Sordi, Alessandro Morettini, Carlo Nozzoli, Loredana Poggesi, Filippo Pieralli, Alessandro Bartoloni, Alessandro Atanasio, Filippo Miselli, Chiara Paoli, Giuseppe G. Loscocco, Andrea Fanelli, Ombretta Para, Andrea Berni, Irene Tassinari, Lorenzo Zammarchi, Laura Maggi, Alessio Mazzoni, Valentina Scotti, Giorgia Falchetti, Danilo Malandrino, Fabio Luise, Giovanni Millotti, Sara Bencini, Manuela Capone, Marie Pierre Piccinni, Francesco Annunziato, Paola Guglielmelli, for the RUXO-COVID Study Group, Francesco Mannelli, Giacomo Coltro, Duccio Fantoni, Miriam Borella, Enrica Ravenda, Benedetta Peruzzi, Roberto Caporale, Lorenzo Cosmi, Francesco Liotta, Letizia Lombardelli, Federica Logiodice, Anna Vanni, Lorenzo Salvati, Chiara Lazzeri, Manuela Bonizzoli, Adriano Peris, Giovanni Cianchi, Alberto Bosi, Michela Pucatti, Paolo Fontanari, Silvia Benemei, Marco Matucci Cerinic, Lucia Turco

**Affiliations:** 1 Center Research Innovation of Myeloproliferative Neoplasms (CRIMM), SOD Hematology, University of Florence and AOU Careggi, Florence, Italy; 2 Internal Medicine Unit 2, AOU Careggi, Florence, Italy; 3 Internal Medicine Unit 1, AOU Careggi, Florence, Italy; 4 Internal Medicine Unit 3, AOU Careggi, Florence, Italy; 5 Intermediate Care Unit COVID-19, AOU Careggi, Florence, Italy; 6 Infectious and Tropical Diseases Unit, AOU Careggi, Florence, Italy; 7 Department of Clinical and Experimental Medicine, University of Florence, Florence, Italy; 8 Cytometry and Immunotherapy Diagnostic Center (CDCI), AOU Careggi, Florence, Italy; 9 Department of Neuromusculoskeletal and Sense Organs, AOU Careggi, Florence, Italy; 10 SOD Hematology, AOU Careggi, Florence, Italy; 11 Hospital Pharmacy, AOU Careggi, Florence, Italy; 12 Department of Anesthesia and Reanimation, AOU Careggi, Florence, Italy; 13 Ethics Committee AVC, AOU Careggi, Florence, Italy; 14 Health Management, AOU Careggi, Florence, Italy

**Keywords:** Infectious diseases, Therapeutics

## Abstract

Overwhelming inflammatory reactions contribute to respiratory distress in patients with COVID-19. Ruxolitinib is a JAK1/JAK2 inhibitor with potent anti-inflammatory properties. We report on a prospective, observational study in 34 patients with COVID-19 who received ruxolitinib on a compassionate-use protocol. Patients had severe pulmonary disease defined by pulmonary infiltrates on imaging and an oxygen saturation ≤ 93% in air and/or PaO2/FiO2 ratio ≤ 300 mmHg. Median age was 80.5 years, and 85.3% had ≥ 2 comorbidities. Median exposure time to ruxolitinib was 13 days, median dose intensity was 20 mg/day. Overall survival by day 28 was 94.1%. Cumulative incidence of clinical improvement of ≥2 points in the ordinal scale was 82.4% (95% confidence interval, 71–93). Clinical improvement was not affected by low-flow versus high-flow oxygen support but was less frequent in patients with PaO2/FiO2 < 200 mmHg. The most frequent adverse events were anemia, urinary tract infections, and thrombocytopenia. Improvement of inflammatory cytokine profile and activated lymphocyte subsets was observed at day 14. In this prospective cohort of aged and high-risk comorbidity patients with severe COVID-19, compassionate-use ruxolitinib was safe and was associated with improvement of pulmonary function and discharge home in 85.3%. Controlled clinical trials are necessary to establish efficacy of ruxolitinib in COVID-19.

## Introduction

Since the first cases reported in December 2019, the pandemic disease COVID-19, due to SARS-CoV-2, has caused more than 376,000 deaths over the world, including 33,500 in Italy [1, 2]. The symptoms caused by SARS-CoV-2 vary from mild respiratory symptoms to severe pneumonia and acute respiratory distress syndrome (ARDS), thromboembolic complications, multiorgan failure, with an overall case fatality ratio of 1–3%, that increased to 14.5 and 27% in patients older than 80 and 85 years, respectively, in two large series [3, 4]. Effective therapies for COVID-19 are not fully established yet, although the RNA polymerase inhibitor remdesivir was recently shown to be superior to placebo in shortening time to recovery and reducing mortality [5–7].

The pathogenesis of COVID-19 involves not only viral replication, but also an overexuberant inflammatory reaction. Treating hyperinflammation may represent a reasonable therapeutic option, ideally in association with antiviral drugs, to limit the extent of tissue damage, especially in the lung, and the rising mortality in COVID-19. Anti-interleukin (IL)-6 and antitumor necrosis factor antibodies [8–10], and the IL-1 receptor antagonist anakinra [11], are under scrutiny to such purpose. Ruxolitinib is a potent JAK1 and JAK2 inhibitor, with good safety profile, that is approved for myelofibrosis [12, 13] and polycythemia vera [14], two myeloproliferative neoplasms characterized by over-inflammation. Ruxolitinib showed clinical activity also in hemophagocytic lymphohistiocytosis [15], and was superior to conventional therapy in acute graft-versus-host disease [16], two largely cytokine-driven diseases. Herein, we report outcomes of patients with severe respiratory manifestations of COVID-19, who received ruxolitinib on a compassionate-use protocol and were enrolled in a prospective observational study.

## Methods

### Study oversight

On April 2, 2020, the Italian Agency for Drug (AIFA) and Istituto Spallanzani approved a treatment protocol study (No. 47) for compassionate use of ruxolitinib in patients with SARS-CoV-2 infection, for up to 28 days. Eligible patients have a positive polymerase chain reaction (PCR) assay on nasopharyngeal swab or lower respiratory tract specimen, and severe COVID-19 manifestations, as defined by presence of pulmonary infiltrates on imaging plus an oxygen saturation ≤ 93% on room air and/or an arterial oxygen partial pressure (PaO2)/fraction of inspired oxygen (FiO2) (P/F) ratio ≤ 300 mmHg. An impaired renal function, as defined by serum creatinine > 2 mg/dl or an estimated creatinine clearance < 30 ml/min, and inability to comply with treatment instructions, was exclusion criteria; patients requiring invasive mechanical ventilation were excluded. The drug supplier, Novartis, had no role in patient selection. A separate observational, prospective, study protocol (RUXO-COVID), was approved by institutional ethic committee in Florence on April 5, 2020 (No. 17104); it allowed the collection of clinical and laboratory data, as well as blood samples for exploratory analyses, in ruxolitinib-eligible patients who had signed a separate, informed, written consent. Novartis had no role in the design of the observational study, data collection, analysis, and interpretation. The study was conducted in accordance with the principles of the declaration of Helsinki and the Good Clinical Practice guidelines.

The study received support from Associazione Italiana per la Ricerca sul Cancro, a no-profit organization (Mynerva project). The draft of the paper was prepared by lead authors, with input from all authors, that vouch for the accuracy and completeness of the data and agree with their interpretation.

### Patients and treatment

Patients were monitored daily regarding health status, oxygen support, ongoing therapies, and adverse events. Blood samples were routinely obtained for blood cell count and chemistry, serum ferritin, D-dimer and C-reactive protein (CRP) determination. Ruxolitinib was administered at a starting daily dose of 5 mg BID; the dose was increased to 10 mg BID after 24–48 h in case there was no improvement of respiratory function and/or oxygen support from baseline, provided no adverse event ≥ grade 3 was observed; further escalation to 25 mg daily was allowed after an additional 48 h. Ruxolitinib was administered with an adaptive approach that allowed patients to receive any other available therapies for COVID-19, as per institutional protocols.

### Study assessment

The following clinical variables were collected: type of oxygen support (noninvasive positive pressure ventilation (NIPPV); high-flow nasal cannulae oxygenation (HFNC); low-flow oxygen); switch to mechanical ventilation; adverse events; hospital discharge; death. After hospital discharge, patients were reached by telephone calls up to day 28, to collect information about their general health status and oxygen support.

Patients were categorized, at baseline (before taking the first ruxolitinib dose), and daily thereafter, according to modified Ordinary Scale for Clinical Improvement, as recommended by the WHO R&D Blueprint expert group [17]. The seven categories are: (1) not hospitalized, with recovery of normal activities; (2) not hospitalized, with residual limitations of normal activities; (3)hospitalized, not requiring oxygen therapy; (4) hospitalized, requiring low-flow oxygen; (5) hospitalized, requiring high-flow oxygen or noninvasive ventilation; (6) hospitalized, requiring intubation and mechanical ventilation, or ventilation plus organ support, or ECMO; (7) death.

Adverse events, irrespective of possible causal association with ruxolitinib, were listed according to the National Cancer Institute Common Terminology Criteria for Adverse Events, v5.0 [18].

### Exploratory laboratory assessment

Blood samples for exploratory analyses were obtained at baseline and days 7 and 14. Immunophenotyping of peripheral blood cells was performed by multiparametric flow cytometry [19] using fluorochrome-conjugated antibodies to lineage-associated surface markers (Supplementary Methods; Table S1). Staining of intracellular cytokines was performed on fixed and permeabilized peripheral blood mononuclear cells, after polyclonal stimulation [13]. Ki67 expression was analyzed using anti-Human Foxp3 Staining Set [20]. The quantitative determination of a panel of 27 serum cytokines was performed by a bead-based multiplex immunoassay [21].

### Statistical methods

This observational study had no sample-size calculation. The analysis included all patients who signed the informed consent form in the period from April 7 to May 8, 2020, before the enrollment was interrupted owing to the rapid decline of hospitalizations for COVID-19. Clinical improvement was described with the use of Kaplan–Meier analysis. Association of baseline characteristics with clinical outcomes was evaluated using Cox proportional hazards regression; since the analysis did not include correction for multiple comparisons of association, results are reported as point estimates and 95% confidence intervals (CI). Differences between longitudinal laboratory values were analyzed using two-tailed paired Student’s *t*-test, or Mann–Whitney *U* test, as appropriate. Analyses were performed with the SPSS software, version 26 (IBM Corp).

## Results

### Patient disposition

In total, 40 patients referring to Azienda Ospedaliera-Universitaria Careggi, Florence, from April 7 to May 8, 2020, fulfilled the criteria for compassionate use of ruxolitinib; they also consented to enter the prospective observational study, whose results are reported herein. Six patients did not receive the treatment because of worsening thrombocytopenia, withdrawal of consent, early shift to intubation (*n* = 1 each), and early death (*n* = 3). Thirty-four patients received at least one dose of ruxolitinib and were included in the analysis; of these, 29 patients (85.3%) were discharged home by the 28-day observation period; 2 patients died, 3 patients were still hospitalized by day 28. Patient disposition is shown in Fig. S1.

### Baseline characteristics of the patients

Table 1 shows baseline demographic, clinical, and laboratory characteristics of the 34 patients who received ruxolitinib. Eighteen patients (52.9%) were male; median age was 80.5 years (interquartile (IQR), 70–85); 52.9% of the patients were 80 years or older. Two or more comorbid conditions were found in 29 patients (85.3%), and included hypertension (*n* = 24; 70.6%), diabetes (*n* = 9; 26.5%), chronic heart disease (*n* = 19; 55.9%), chronic pulmonary disease (*n* = 10; 29.4%), chronic kidney disease (*n* = 1; 2.9%), cancer (*n* = 10; 29.4%), neurologic impairment (*n* = 15; 44.1%), autoimmune disease (*n* = 5; 14.7%). The median comorbidity Charlson index was 6 (IQR, 4.2–6.0). Ten patients (29.4%) were active smokers or had a history of smoking.

**Table 1:** Baseline demographic, clinical and laboratory characteristics of the patients who received ruxolitinib.

Characteristics	Total (*n* = 34)
Sex, male—No. (%)	18 (52.9)
Median age (IQR), years	80.5 (70–85)
Age category—No. (%)	
<60 years	4 (11.8)
60–<80 years	12 (35.3)
≥80 years	18 (52.9)
Comorbidities—No. (%)	
Hypertension	24 (70.6)
Diabetes	9 (26.5)
Chronic heart disease	19 (55.9)
Chronic pulmonary disease	10 (29.4)
Chronic kidney disease	1 (2.9)
Cancer	10 (29.4)
Neurologic impairment	15 (44.1)
Autoimmune disease	5 (14.7)
One comorbidity	5 (14.7)
Two or more comorbidities	29 (85.3)
Smoking habit	10 (29.5)
Charlson Comorbidity Index, median (IQR)	6.0 (4.2–6.0)
Median duration (IQR) of symptoms before ruxolitinib—days	8.0 (3.5–11.5)
Stage according to the seven-category ordinal scale (mod)—score No. (%)	
5	17 (50.0)
4	16 (47.1)
3	1 (2.9)
Oxygen support—No. (%)	
High-flow oxygen	
Noninvasive positive pressure ventilation (NIPPV), or high-flow nasal cannula (HFNC)	7 (20.6)
Standard high-flow oxygen (FiO2 > 40%)	10 (29.4)
Low-flow oxygen	16 (47.1)
Ambient air	1 (2.9)
Median oxygen saturation on room air (IQR)—(of 29 patients with available information)	91 (89–93)
Median PaO2/FiO2 (P/F) value (IQR)	240 (128–277)
SOFA score—points, No. (%)	
0–1	1 (2.9)
2–3	23 (67.6)
4–5	10 (29.4)
>5	0
PaO2/FiO2 (P/F), No. (%)	
≥300	4 (11.8)
≥200 < 300	15 (44.1)
≥100 < 200	10 (29.4)
<100	5 (14.7)
Laboratory characteristics, median (IQR)	
Leukocytes—×10^9^/l	5.57 (4.58–8.59)
Lymphocytes—×10^9^/l	0.78 (0.65–1.2)
Hemoglobin—g/l	122 (108–129)
Platelets—×10^9^/l	184 (160–256)
D-dimer—ng/m	1031 (750–1478)
Ferritin—mg/l	639 (349–838)
C-reactive protein—mg/l	73 (39–111)
Concomitant medications for COVID-19—No. (%)	
Lopinavir/ritonavir	12 (35.3)
Darunavir/cobicistat	8 (23.5)
Remdesivir	1 (2.9)
Hydroxychloroquine	31 (91.2)
Heparin	34 (100)
Corticosteroids	10 (29.4)
Antibiotics	26 (76.5)
Antifungal	2 (5.9)

The median number of days between symptoms onset and start of ruxolitinib was 8 (IQR, 3.5–11.5). According to the ordinal scale, 17 patients (50.0%) met criteria for category 5, of which 7 patients (41.2%) required NIPPV/HFNC and 10 patients (58.8%) required standard high-flow oxygen (FiO2 > 40%); 16 patients (47.1%) met criteria for category 4, and received low-flow oxygen; one patient (2.9%) had dyspnea but maintained a partial oxygen saturation of 93% while breathing ambient air (category 3). The median oxygen saturation on room air was 91% (IQR, 89–93%), and the median P/F ratio was 240 mmHg (IQR, 128–277) [22]. Four patients (11.8%) had a P/F ratio > 300 (range, 301–333), 15 patients (44.1%) were ≥200 < 300, 10 patients (29.4%) were ≥100 < 200, 5 patients (14.7%) were <100. The distribution of patients according to SOFA score was as follows: 1 patient (2.9%) was 0–1 point, 23 patients (67.6%) 2–3 points, 10 patients (29.4%) 4–5 points [23].

Concomitant therapies were antiviral drugs in 21 patients (61.8%), of which only 1 received remdesivir; hydroxychloroquine in 31 patients (91.2%); antimicrobials in 26 patients (76.5%); corticosteroids in 10 patients (29.4%). All patients received prophylactic doses of subcutaneous enoxaparin.

Laboratory investigations showed reduced median lymphocyte count (0.78 × 10^9^/l; IQR, 0.65–1.2) and elevated median levels of D-dimer (1031 ng/ml; IQR, 750–1478), serum ferritin (639 mg/l; IQR, 349–838), and CRP (73 mg/l; IQR, 40–116). Three patients (8.8%) had hemoglobin < 100 g/l, one patient had thrombocytopenia (88 × 10^9^/l).

### Clinical outcomes during treatment with ruxolitinib

**Fig. 1:**
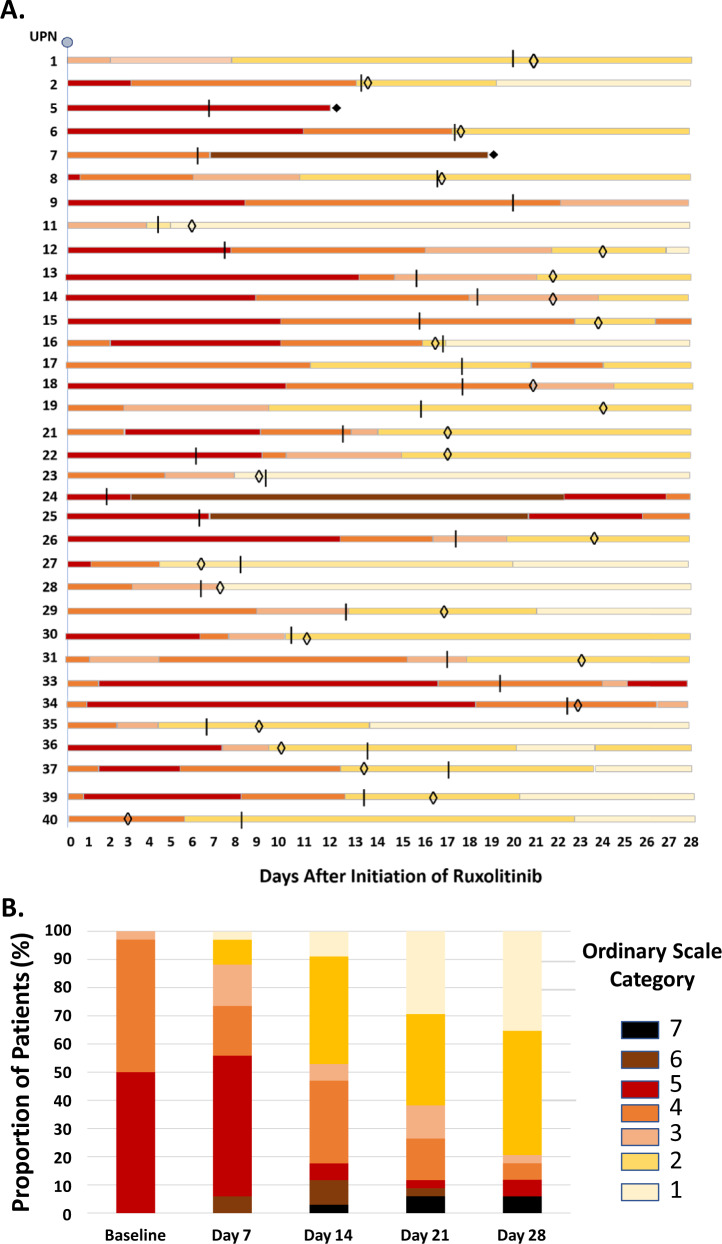
Changes in the category of the ordinal scale in individual patients, and in the full cohort of patients. Each patient is represented as a colored line, where each color indicates the category of the ordinal scale to which the patient belongs, from baseline (day 0, day of first dose of ruxolitinib) to day 28. The vertical bars indicate the last day of treatment with full dose of Ruxolitinib. A solid diamond indicates that the patient died. Patients were monitored daily while hospitalized, and reached by telephone calls every 2–3 days after being discharged. The day of discharge is indicated by an open diamond (**a**). The cumulative distribution of patients in the individual categories of the ordinal scale, at weekly intervals, is shown in (**b**).

To describe effects of treatment, we used the definition of clinical improvement as a decrease of ≥2 points in the ordinal scale, from first dose of ruxolitinib up to day 28. Individual patients’ changes in the ordinal scale, and distribution of patients in different categories by time intervals, are shown in Fig. 1. A total of 29 patients (85.3%) met criteria for clinical improvement; of the 5 patients who did not, 2 patients were category 4, 3 patients were category 5. Fourteen of the 16 patients (87.5%) who were receiving low-flow supplemental oxygen (category 4) showed clinical improvement; of the 2 who did not, 1 patient stopped ruxolitinib on day 7 because of no improvement and died on day 20 due to bacterial sepsis, 1 patient stopped ruxolitinib on day 18 because of *ab ingestis* pneumonia and was still hospitalized by day 28. Clinical improvement was observed in 14 of 17 patients (82.4%) receiving high-flow oxygen (category 5); of the 3 who did not, 1 patient stopped ruxolitinib on day 7 because of no improvement and died on day 12 due to cardiorespiratory failure; 2 patients stopped ruxolitinib on days 2 and 3 because they required intubation, and were still hospitalized by day 28.

**Fig. 2:**
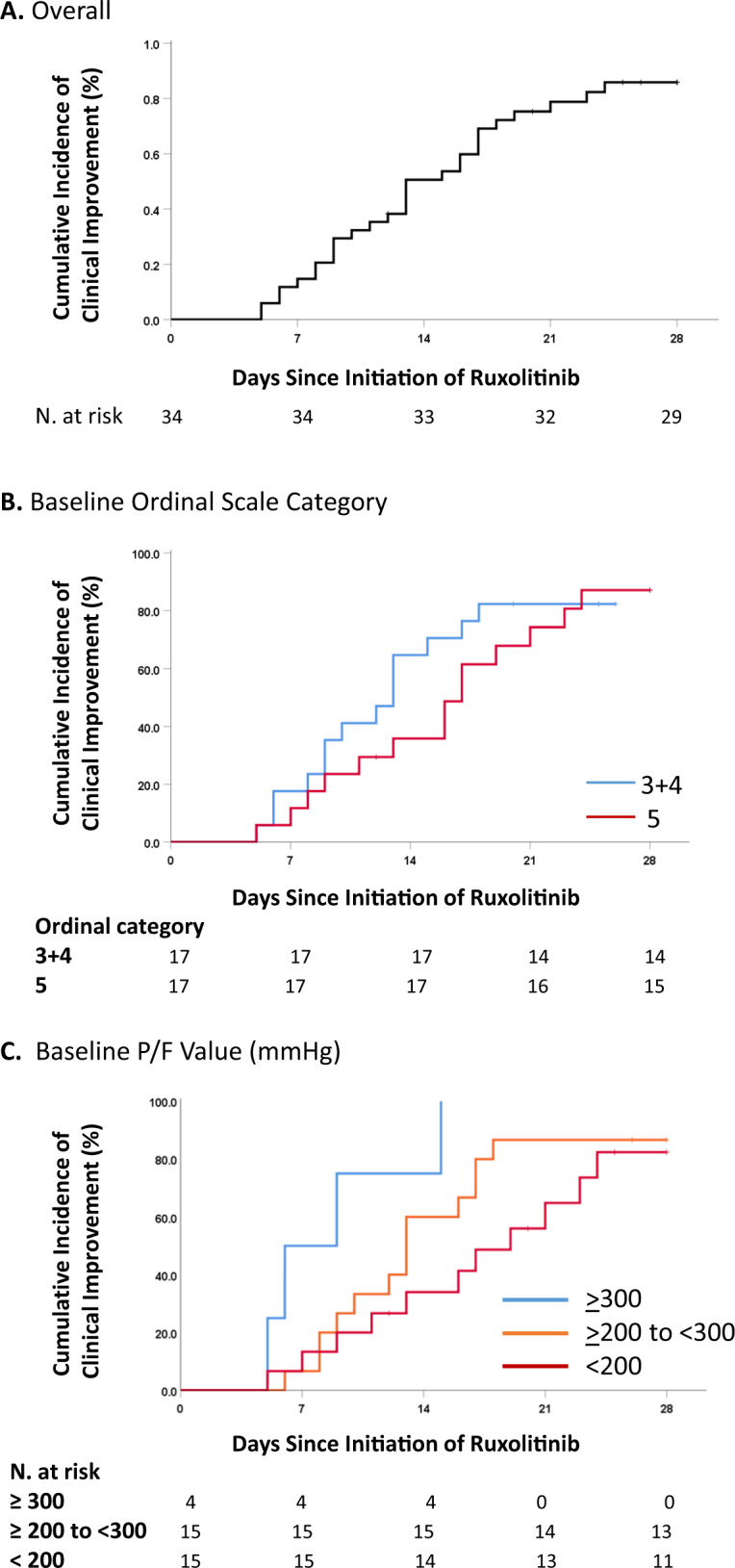
Cumulative incidence of clinical improvement from baseline to day 28. The data are shown for the full cohort of patients (**a**), for patients in the full cohort stratified according to the ordinal scale category at baseline (**b**), and for patients in the full cohort stratified according to the arterial oxygen partial pressure (PaO2)/fraction of inspired oxygen (FiO2) (P/F ratio) at baseline (**c**).

The cumulative incidence of clinical improvement was 82.4% (95% CI, 71–93) (Fig. 2a). Clinical improvement was not affected by need of high-flow oxygen support (category 5) (hazard ratio for clinical improvement, as compared to category 3 + 4, was 0.74; 95% CI, 0.35–1.57) (Fig. 2b). Conversely, clinical improvement was less frequent among patients with more severe respiratory impairment: as compared to patients with P/F ≥ 300 mmHg, the hazard ratio was 0.31 (95% CI, 0.1–1.0) for patients with P/F ratio <300 ≥ 200, and 0.20 (95% CI, 0.06–0.67) for patients with P/F ratio < 200 (Fig. 2c). Sex, age, comorbidities, duration of symptoms, use of antiviral agents, and laboratory abnormalities were not associated with clinical improvement (Table S2).

### Safety

The median duration of exposure to ruxolitinib was 13 days (IQR, 7.3–16.8). The median dose intensity of ruxolitinib was 20 mg per day (IQR, 20–25); the maximum dose of ruxolitinib was 10 mg/day in 5 patients (14.7%), 15 mg/day in 2 patients (5.9%), 20 mg/day in 17 patients (50.0%), and 25 mg/day in 10 patients (29.4%). Discontinuation of treatment occurred in five patients (14.7%); reason was clinical deterioration requiring intubation (*n* = 2; 5.9%), *ab ingestis* pneumonia (*n* = 1; 2.9%), death (*n* = 2; 5.9%).

Adverse events, or worsening of preexisting laboratory abnormality, developed in 28 patients (82.3%), including grade 3 in 13 patients (38.2%); in no case they led to drug discontinuation. The most common adverse events of any grade were anemia, urinary tract infection, increase of creatinine, thrombocytopenia, increase of aminotransferases. Anemia developed in 6 patients (17.6%; 1 grade 3) and worsened from baseline in 13 patients (38.2%; grade 3 in 10 patients, 29.4%) (Table 2); 8 patients (23.5%) required two red blood cell units. Thrombocytopenia developed in four patients, all grade 1; one patient (2.9%) had worsening of baseline thrombocytopenia to grade 3, but did not require platelet transfusion nor ruxolitinib dose reduction. Three thrombotic events were recorded: pulmonary embolism, brachial vein thrombosis following positioning of venous catheter, and peripheral arterial thrombosis.

**Table 2:** Summary of adverse events up to day 28.

Event or abnormalities	Number of patients (percent)		
	Present before initiation of treatment	Developed/worsened during treatment	
		Any grade	Grade 3
Any adverse event	33 (97.1)	28 (82.3)	13 (38.2%)
Anemia	25 (73.5)	19 (55.9)	10 (29.4)
Thrombocytopenia	8 (2.3)	5 (14.7)	1 (2.9)
Neutropenia	1 (2.9)	2 (5.9)	0
Aminotransferase increased	10 (29.4)	5 (14.7)	0
Creatinine increased	14 (41.2)	8 (23.5)	3 (8.8)
Bleeding	1 (2.9)	3 (8.8)	1 (2.9)
Urinary tract infection	2 (5.9)	10 (29.4)	0
Sepsis	0	2 (8.8)	1 (2.9%)
GI infection	0	3 (8.8)	0
Bacterial pneumonia	0	2 (5.9)	0
Arrhythmia	3 (8.8)	3 (8.8)	0
Xanthelasma	1 (2.9)	1 (2.9)	0
Stroke	1 (2.9)	1 (2.9)	1 (2.9)
Acute pancreatitis	0	1 (2.9)	0
Thrombosis	0	2 (5.9)	1 (2.9)

### SARS-CoV-2 viral status

A total of 23 patients (67.6%) resulted PCR-negative on double check of upper respiratory tract swab at a median of 21 days (IQR, 17–26) after initiation of treatment with ruxolitinib. Exhibiting negative PCR assay was not affected by use of antivirals (HR, 0.58; CI, 0.1–3.6).

### Exploratory measures

**Fig. 3:**
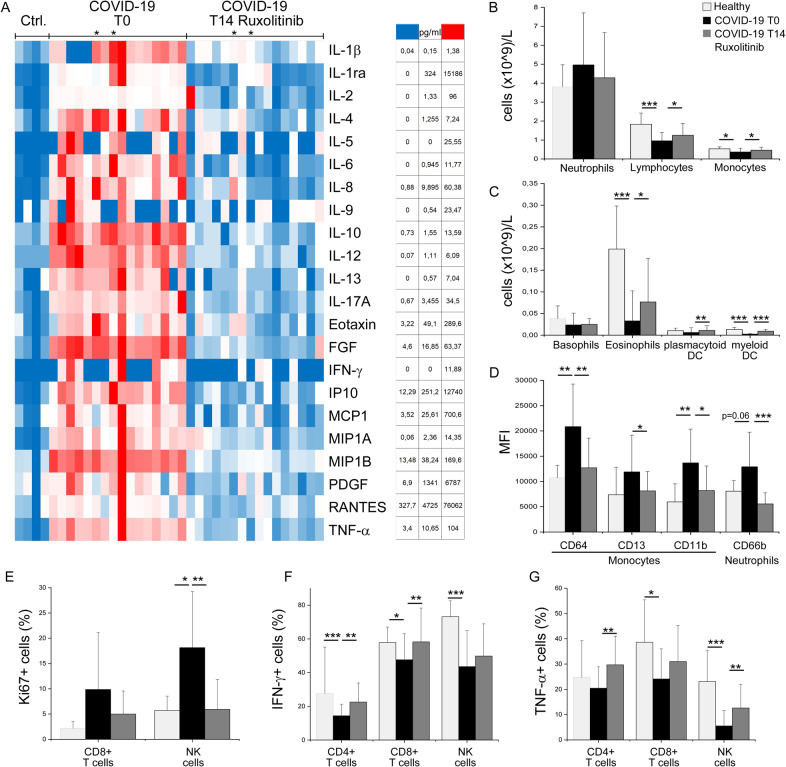
Changes in peripheral blood mononuclear cell subsets and serum cytokine levels at day 14 in COVID-19 patients compared to levels at baseline and normal subjects. The absolute count of peripheral blood cell subsets, analyzed by flow cytometry, was measured at baseline (T0, black columns) and at day 14 (T14, dark gray columns) since initiation of ruxolitinib. Columns represent mean value (±SD) of neutrophils, lymphocytes, monocytes, basophils, eosinophils, plasmacytoid, and myeloid dendritic cells (DC). Data were obtained from 16 COVID-19 patients receiving ruxolitinib, and healthy donors (*n* = 8) (**a**, **b**). The activation markers CD64, CD13, and CD11b (on monocytes), and CD66b (on granulocytes), were analyzed by flow cytometry in the same set of samples; results are expressed as the mean value (±SD) of mean fluorescence intensity (MFI) (**c**). The frequency of Ki67-positive cells, expressed as the Mean (±SD), was obtained from analysis of isolated peripheral blood mononuclear cells of COVID-19 patients (*n* = 13), collected at T0 and T14, and healthy donors (*n* = 6) as control (**d**). The frequency of IFN-gamma of TNF-alpha positive cells, obtained from analysis of isolated peripheral blood mononuclear cells after in vitro polyclonal stimulation, is expressed as mean (+SD). Data refer to 14 COVID-19 patients, and 12 healthy donors, as control (**e**, **f**). **g** Heatmap of serum concentration (pg/ml) of the indicated cytokines and chemokines in healthy controls (*n* = 4) and COVID-19 patients (*n* = 16), who were evaluated at baseline (T0) and at day 14 (T14) since initiation of ruxolitinib. Only two patients, indicated by an asterisk, were receiving corticosteroids concurrently with ruxolitinib, in the first 7 days of treatment. The color scale ranges from blue (lower concentration) to red (higher concentration) for each analyte. *p < 0.05, **p < 0.001, ***p < 0.001, as indicated by the bars.

**Fig. 4:**
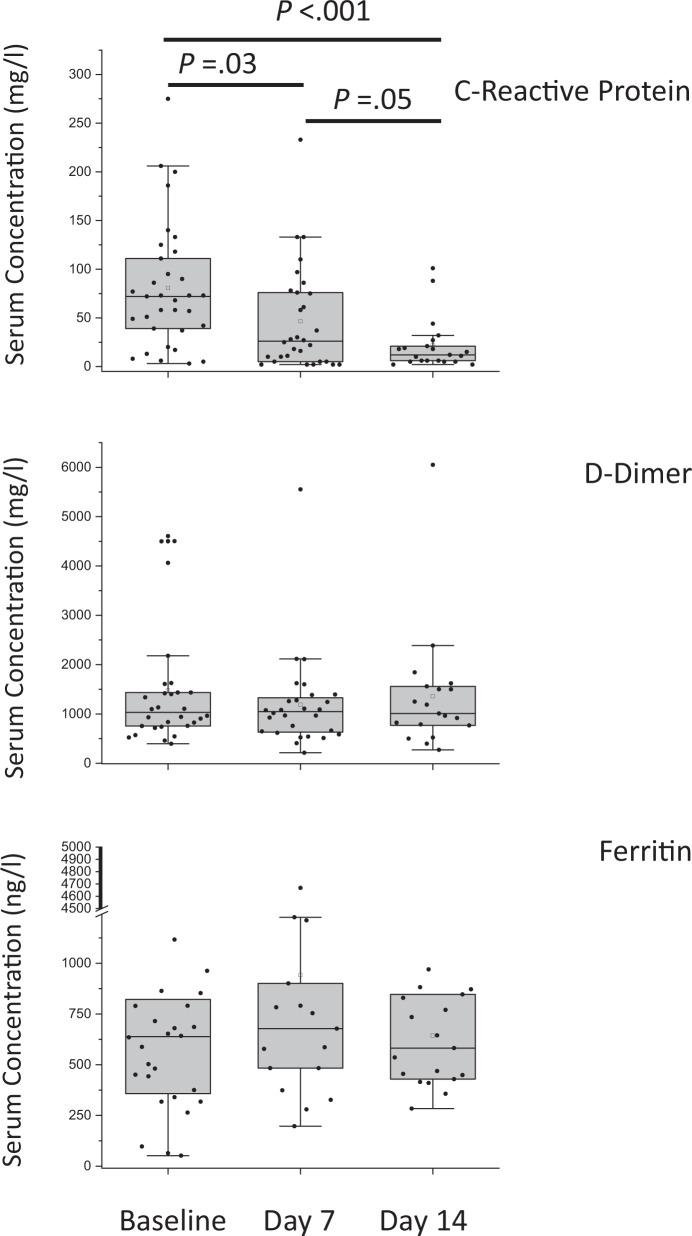
Changes in C-reactive protein, D-dimer, and ferritin levels at days 7 and 14 in COVID-19 patients, compared to levels at baseline. The plasma levels of C-reactive protein and D-dimer, and serum levels of ferritin, were measured at baseline and at days 7 and 14 since initiation of ruxolitinib. Individual values are presented as well as the mean value ± SD. Statistically significant differences are shown on top.

In patients receiving ruxolitinib, the absolute count of lymphocytes, monocytes, eosinophils, and myeloid and plasmacytoid dendritic cells, that were all significantly decreased at baseline [24], resulted largely restored by day 14, as it was the abnormally increased expression of markers of activation of neutrophils (CD66b) and monocytes (CD64, CD13, CD64) (Fig. 3a–c; Table S3). The frequency of innate (CD3−CD16+) NK cytotoxic cells expressing the cell-cycling marker Ki67, which rapidly expand in response to viral infection [20], also returned to normal levels by day 14, with similar trend for adaptive, cytotoxic CD3+CD8+ T cells (Fig. 3d). An improvement in the frequencies of IFN-γ producing T cells and TNF-α producing NK cells was documented (Fig. 3e, f) [13]. Serum levels of a panel of 27 cytokines and chemokines resulted markedly increased at baseline, compared to control subjects, with few exceptions (IL1Ra, IL-9, monocyte chemoattractant protein 1 (MCP1), PDGF, Rantes); among the most dysregulated, we noticed IL-6 (89.6-fold), interferon gamma-induced protein-10 (87.5-fold), and MCP1 (54.3-fold) (Table S4). All resulted markedly decreased toward normal levels by day 14 of treatment (Fig. 3g). Longitudinal analysis showed that CRP levels significantly decreased from a baseline median level of 72 mg/l (IQR, 39–111) to 26 mg/l (IQR, 5–76; *p* = 0.03) by day 7 and normalized by day 14 (12 mg/l, IQR, 6–21; *p* < 0.001); no significant change was observed for D-dimer and ferritin (Fig. 4).

## Discussion

There is evidence that the pneumonia caused by SARS-CoV-2, representing the leading cause of death in patients with COVID-19, involves a systemic hyperinflammatory reaction [25, 26]. The latter contributes potently to the lung damage caused by the entry of the virus in respiratory epithelium, mediated by the receptor angiotensin converting enzyme-2 (ACE2) [27], and eventually results in acute, potentially fatal, respiratory distress syndrome (ARDS). Similarities between this localized, overwhelming inflammatory reaction, and diseases associated with a systemic cytokine release storm, such as the secondary haemophagocytic lymphohistiocytosis [28], have been highlighted [29]. Notably, children with history of SARS-CoV-2 infection may develop Kawasaki-like syndrome, a rare, largely cytokine-mediated, disease [30]. Hyperinflammation may also affect the vascular system, contributing to thrombotic events in pulmonary vessels and systemic circulation, that was reported at unusual rate [31–34]. Plasma levels of a vast array of inflammatory cytokines, some of which were associated with severity of clinical manifestations and more advanced disease requiring intensive care, are markedly elevated in patients with COVID-19 [35]; a condition of IL-6-dependent, impaired, immune cell cytotoxicity may contribute to abnormal immunoregulation caused by SARS-CoV-2 infection [24]. Therefore, targeting the host inflammatory response might play an important role in dampening hyperinflammation and reducing lung damage in patients with COVID-19, especially when the ARDS has not yet progressed to terminal stages of pulmonary failure requiring mechanical ventilation [26].

No specific therapy for COVID-19 still exists, management largely consisting of supportive care, and most patients received off-label or compassionate-use therapies including antiretrovirals, antiparasitic agents, anti-inflammatory compounds, and convalescent plasma. Among antivirals, the most widely used are remdesivir and lopinavir/ritonavir. Recent controlled data indicated improved outcome with remdesivir [5–7, 36], and a preliminary report from the ACCT-1 trial suggested that remdesivir was superior to placebo in shortening time to recovery [5]. Conversely, a randomized trial on 199 patients with severe disease who were treated with lopinavir/ritonavir failed to demonstrate any benefit in comparison to standard of care, and the NIH recommended against its use due to unfavorable pharmacodynamics and lack of proven clinical efficacy [37]. Hydroxychloroquine was largely used in the early period of COVID-19 pandemic owing to its in vitro activity against the virus and relatively safe profile. However, an observational study of 811 patients treated with hydroxychloroquine failed to show any clinical benefit compared with standard of care [38], and a systematic review raised concerns about the quality of published studies [39]. Interim results of the Solidarity Trial showed no significant reduction in mortality of hospitalized COVID-19 patients treated with either hydroxychloroquine or lopinavir/ritonavir compared to standard of care, and these arms of the study were discontinued.

Preliminary evidences also support potential efficacy of dugs with anti-inflammatory properties, some of which are currently in clinical trials, including tocilizumab (a monoclonal antibody blocking the anti-IL-6 receptor; e.g., NCT04346355) and anakinra (a recombinant IL-1 receptor antagonist; e.g., NCT04364009), or are potential candidates, such as baricitinib (a JAK1 and JAK2 inhibitor) [29]. In addition to suppressing cytokine signaling and preventing the emergence of cytokine storm, baricitinib may interrupt the passage and intracellular assembly of SARS-CoV-2, according to recent data [40]. An open-label trial reported encouraging results of baricitinib in terms of safety, improvement of clinical conditions and reduction of progression to more severe forms [41]. However, baricitinib should be used with caution in patients with thrombotic risk factors because of the increased risk of deep venous thrombosis and pulmonary embolism, and clinical experience in patients ≥ 75 years is very limited. As regards the use of corticosteroids in COVID-19, there are conflicting findings and recommendations. The main issues against the use of steroids are the risk of prolonged viral shedding [42] and secondary bacterial infections [43]. Conversely, in other studies no impact of corticosteroid therapy on viral RNA shedding was found, and low-dose corticosteroid did not affect viral RNA clearance [44]. Available clinical evidence did not support a benefit of corticosteroids in the treatment of respiratory infection due to RSV, influenza, SARS-CoV, or MERS-CoV [45]. On the other hand, preliminary results from the RECOVERY trial showed that dexamethasone reduced deaths in patients receiving either invasive [RR, 0.65; 95% CI, 0.51–0.82] and noninvasive [RR, 0.80; 95% CI, 0.70–0.92] mechanical ventilation [46]; furthermore, corticosteroid treatment was associated with a reduced risk of death in patients who developed ARDS [47, 48]. Corticosteroid treatment is “a double edged sword” in COVID-19 [49], and randomized, controlled trials are definitely needed.

Ruxolitinib is a JAK1 and JAK2 inhibitor with potent anti-inflammatory properties and excellent safety profile, that is approved for the treatment of myelofibrosis [12, 13] and polycythemia vera [14, 50, 51]; remarkably, ruxolitinib proved to be efficacious in conditions characterized by exaggerated release of inflammatory cytokines and activation of immunocompetent cells, such as the hemophagocytic lymphohistiocytosis [15] and the graft-versus-host disease in recipients of allogeneic hematopoietic stem cell transplantation [16]. We describe here results of a prospective, observational study in 34 patients with severe pulmonary manifestations of COVID-19, not requiring mechanical ventilation, who received compassionate-use ruxolitinib within a treatment protocol approved by Italian Agency for Drugs. The study population was uniquely represented by old (median age, 80.5 years), high-risk comorbid, subjects, mirroring the epidemiology of late hospitalization for SARS-CoV-2-infected patients coming from Italian Extended Care Units. We observed clinical improvement, as defined based on ordinal scale, in 85.3% of patients after a median of 13 days, with mortality rate of 5.9% by 28 days. This short-term treatment with ruxolitinib (median treatment duration was 13 days) resulted well tolerated, with few grade 3 events, and no new safety signals were detected. Of note, 67.6% of the patients exhibited confirmed, negative PCR swab assays at the end of study period, irrespective of having received antiviral agents, suggesting that ruxolitinib does not prevent viral clearance. Anti-inflammatory and immunomodulatory activities of ruxolitinib were documented by normalization of blood cell subsets, dampening of inflammatory cell activation, and decrease of inflammatory cytokines and CRP levels, providing clues to the immunoregulatory effects of ruxolitinib in COVID-19 patients. Dampening of inflammation induced by ruxolitinib treatment may favorably impact also on the increased rate of thrombosis associated with COVID-19. The coagulopathy associated with COVID-19 partially overlaps with other coagulopathies, such as sepsis-induced coagulopathy or disseminated intravascular coagulation, although it does not perfectly match any of them [34]. It remains to be determined if such hypercoagulability condition is caused by activation of innate immune response with complement-mediated microthrombotic manifestations [34] and/or by an endotheliopathy, possibly mediated by interaction of SARS-Cov-2 with ACE2 receptors on endothelial cells [52], eventually exacerbated by the elevated levels of inflammatory cytokines and chemokines.

This study has intrinsic limitations that preclude full interpretation of results, including the small size of the cohort, the heterogeneity of concomitant medications, the inability to perform multivariable analysis, and the lack of a randomized control group, that was not feasible owing to the availability of ruxolitinib on a compassionate-use protocol. Furthermore, the study was prematurely interrupted due to the decline of COVID-19 hospitalization that occurred in the most recent weeks after the lock-down period in Italy. However, the extent of favorable outcomes observed herein is noteworthy, especially considering the advanced age (median, 80.5 years) and the characteristics of this highly comorbid population. Age, and associated comorbidity, emerged as one major risk factor for severe complications and deaths in individuals with COVID-19 [53]. In an Italian study, 42.2% of those who died were older than 80 years, as compared to 32.4% if aged 70–79, and 11.2% if aged 60 years and less [54]. By way of comparison, in a randomized trial of lopinavir-ritonavir in younger patients (median age, 58 years) the 28-day mortality was 22% [55], while in two recent randomized trials using remdesivir in patients with median age of 59 and 62, respectively, the day 14 mortality was 7.1% [5] and 8–11% [6].

Results from two other clinical studies with ruxolitinib were reported recently, plus a few single cases [56, 57]. In the study of La Rosée et al. [58], 14 COVID-19 patients with evidence of severe hyperinflammation, based on a newly developed COVID-19 Inflammation Score, received ruxolitinib over a median of 9 days and a median cumulative dose of 135 mg (approximately, 15 mg day). Evidence of reduced hyperinflammatory status was obtained in 12 patients, and sustained clinical improvement was reported in 11 patients, without any notable toxicity. In the study by Cao et al. [59], a faster clinical improvement was observed in 20 patients receiving ruxolitinib, compared to control group, although the rate of overall clinical improvement was similar. Treatment was well tolerated.

Taking into an account the role of hyperinflammation in the pathogenesis of COVID-19 pneumonia and these preliminary clinical reports [56–59], current findings support the development of controlled trials of ruxolitinib in patients with severe pulmonary manifestations of COVID-19, with the aim to control hyperinflammation and mitigate the progression of the disease. A phase 3, multicenter, double-blind, placebo-controlled study, randomizing patients with COVID-19, who are not in need of mechanical ventilation, to ruxolitinib (5 mg twice daily) or placebo, in addition to standard of care, is ongoing (NCT04362137); the primary endpoint will be comparison of efficacy, including death, progression of respiratory failure and need of intensive care, between the two arms by day 29. It should also be noted that patients requiring mechanical ventilation ab initio were excluded from our study, and efficacy in that setting cannot be borrowed. Indeed, the fact that a P/F ratio < 200 mmHg was negatively associated with clinical improvement in our patients suggests incremental benefits of early treatment. In addition, a separate phase 3, randomized, double-blind, placebo-controlled study will assess the efficacy and safety of ruxolitinib at two different dosages (5 and 15 mg twice daily) in patients with COVID-19-associated ARDS who require mechanical ventilation (NCT04377620). Although our study did not compare different doses of ruxolitinib, due to the adapted incremental dose adjustment, current data support the safety and efficacy of a dose similar to that used in a phase 3 study in glucocorticoid-refractory, acute graft-versus-host disease (the median dose intensity in our study was 20 mg daily) [16]. It is hoped that those controlled trials will contribute to definitely establish whether, and to what extent, the anti-inflammatory activity of ruxolitinib may contribute to reduce mortality from COVID-19, helping “the dust to settle” [60].

## Supplementary information

Supplementary appendix
