# Supplementary material for: Compassionate use of JAK1/2 inhibitor ruxolitinib for severe COVID-19: a prospective observational study
Source: Leukemia. 2020 Aug 19;35(4):1121–33. doi: 10.1038/s41375-020-01018-y (PMC7437386; doi:10.1038/s41375-020-01018-y)
Supplement: Supplementary file 1 — Supplementary appendix [file 41375_2020_1018_MOESM1_ESM.pdf]

SUPPLEMENTARY APPENDIX

To: Alessandro M Vannucchi et al. Compassionate Use of JAK1/2 Inhibitor Ruxolitinib for Severe Covid-19.

Table of contents

RUXO-COVID Study group Participants. page 3

Methods. page 5

Table S1: List of all fluorochrome mAbs used for flow cytometric analysis. page 7

Table S2: Baseline predictors of clinical improvement. page 8

Table S3: Longitudinal evaluation of leukocyte subsets. page 9

Table S4: Longitudinal evaluations of serum inflammatory cytokine levels. page 10

Figure S1. Patient Disposition. page 11

References. page 12

RUXO-COVID Study Group Participants.

Participant	Institution
**Alessandro Maria Vannucchi**	Center Research Innovation of Myeloproliferative Neoplasms (CRIMM), SOD Hematology, University of Florence and AOU Careggi, Florence, Italy
**Benedetta Sordi**	Center Research Innovation of Myeloproliferative Neoplasms (CRIMM), SOD Hematology, University of Florence and AOU Careggi, Florence, Italy
**Alessandro Morettini**	Internal Medicine Unit 2, AOU Careggi, Florence, Italy
**Carlo Nozzoli**	Internal Medicine Unit 1, AOU Careggi, Florence, Italy
**Loredana Poggesi**	Internal Medicine Unit 3, AOU Careggi, Florence, Italy
**Filippo Pieralli**	Intermediate Care Unit Covid-19, AOU Careggi, Florence, Italy
**Alessandro Bartoloni**	Infectious and Tropical Diseases Unit, AOU Careggi, Florence, Italy
**Alessandro Atanasio**	Center Research Innovation of Myeloproliferative Neoplasms (CRIMM), SOD Hematology, University of Florence and AOU Careggi, Florence, Italy
**Filippo Miselli**	Center Research Innovation of Myeloproliferative Neoplasms (CRIMM), SOD Hematology, University of Florence and AOU Careggi, Florence, Italy
**Chiara Paoli**	Center Research Innovation of Myeloproliferative Neoplasms (CRIMM), SOD Hematology, University of Florence and AOU Careggi, Florence, Italy
**Giuseppe G. Loscocco**	Center Research Innovation of Myeloproliferative Neoplasms (CRIMM), SOD Hematology, University of Florence and AOU Careggi, Florence, Italy
**Andrea Fanelli**	Internal Medicine Unit 2, AOU Careggi, Florence, Italy
**Ombretta Para**	Internal Medicine Unit 1, AOU Careggi, Florence, Italy
**Andrea Berni**	Internal Medicine Unit 3, AOU Careggi, Florence, Italy
**Irene Tassinari**	Intermediate Care Unit Covid-19, AOU Careggi, Florence, Italy
**Lorenzo Zammarchi**	Infectious and Tropical Diseases Unit, AOU Careggi, Florence, Italy
**Laura Maggi**	Department of Clinical and Experimental Medicine, University of Florence, Florence, Italy
**Alessio Mazzoni**	Department of Clinical and Experimental Medicine, University of Florence, Florence, Italy
**Valentina Scotti**	Internal Medicine Unit 2, AOU Careggi, Florence, Italy
**Danilo Malandrino**	Internal Medicine Unit 3, AOU Careggi, Florence, Italy
**Fabio Luise**	Intermediate Care Unit Covid-19, AOU Careggi, Florence, Italy
**Giovanni Millotti**	Infectious and Tropical Diseases Unit, AOU Careggi, Florence, Italy
**Sara Bencini**	Cytometry and Immunotherapy Diagnostic Center (CDCI), AOU Careggi, Florence, Italy
**Duccio Fantoni**	Center Research Innovation of Myeloproliferative Neoplasms (CRIMM), SOD Hematology, University of Florence and AOU Careggi, Florence, Italy
**Giorgia Falchetti**	Internal Medicine Unit 2, AOU Careggi, Florence, Italy
**Miriam Borella**	Center Research Innovation of Myeloproliferative Neoplasms (CRIMM), SOD Hematology, University of Florence and AOU Careggi, Florence, Italy
**Enrica Ravenda**	Center Research Innovation of Myeloproliferative Neoplasms (CRIMM), SOD Hematology, University of Florence and AOU Careggi, Florence, Italy
**Manuela Capone**	Department of Clinical and Experimental Medicine, University of Florence, Florence, Italy

**Marie Pierre Piccinni**	Department of Clinical and Experimental Medicine, University of Florence, Florence, Italy
**Francesco Annunziato**	Department of Clinical and Experimental Medicine, University of Florence, Florence, Italy; Cytometry and Immunotherapy Diagnostic Center (CDCI), AOU Careggi, Florence, Italy
**Paola Guglielmelli**	Center Research Innovation of Myeloproliferative Neoplasms (CRIMM), SOD Hematology, University of Florence and AOU Careggi, Florence, Italy
**Francesco Mannelli**	Center Research Innovation of Myeloproliferative Neoplasms (CRIMM), SOD Hematology, University of Florence and AOU Careggi, Florence, Italy
**Giacomo Coltro**	Center Research Innovation of Myeloproliferative Neoplasms (CRIMM), SOD Hematology, University of Florence and AOU Careggi, Florence, Italy
**Benedetta Peruzzi**	Cytometry and Immunotherapy Diagnostic Center (CDCI), AOU Careggi, Florence, Italy
**Roberto Caporale**	Cytometry and Immunotherapy Diagnostic Center (CDCI), AOU Careggi, Florence, Italy
**Lorenzo Cosmi**	Department of Clinical and Experimental Medicine, University of Florence, Florence, Italy
**Francesco Liotta**	Department of Clinical and Experimental Medicine, University of Florence, Florence, Italy
**Letizia Lombardelli**	Department of Clinical and Experimental Medicine, University of Florence, Florence, Italy
**Federica Logiodice**	Department of Clinical and Experimental Medicine, University of Florence, Florence, Italy
**Anna Vanni**	Department of Clinical and Experimental Medicine, University of Florence, Florence, Italy
**Lorenzo Salvati**	Department of Clinical and Experimental Medicine, University of Florence, Florence, Italy
**Chiara Lazzeri**	Department of Neuromuscoloskeletric and sense organs, AOU Careggi, Florence, Italy
**Manuela Bonizzoli**	Department of Neuromuscoloskeletric and sense organs, AOU Careggi, Florence, Italy
**Adriano Peris**	Department of Neuromuscoloskeletric and sense organs, AOU Careggi, Florence, Italy
**Giovanni Cianchi**	Department of Neuromuscoloskeletric and sense organs, AOU Careggi, Florence, Italy
**Alberto Bosi**	SOD Hematology, AOU Careggi, Florence, Italy
**Michela Pucatti**	Hospital Pharmacy, AOU Careggi, Florence, Italy
**Fontanari Paolo**	Department of Anesthesia and Reanimation, AOU Careggi, Florence, Italy
**Silvia Benemei**	Ethics Committee AVC, AOU Careggi, Florence, Italy
**Marco Matucci Cerinic**	Ethics Committee AVC, AOU Careggi, Florence, Italy
**Lucia Turco**	Health management, AOU Careggi, Florence, Italy

Supplementary Methods

Immunophenotyping and intracellular staining by multiparametric flow cytometric analysis

All flow cytometric analysis were performed following published guidelines[1]. Samples were acquired on a BD LSR II flow cytometer or FACS Canto II (BD Biosciences). List of all fluorochrome-conjugated mAbs is reported in Table X (supplementary). Different leukocytes cell subsets were identified by staining lineage surface markers on whole blood, followed by red blood cells lysis prior to FACS analysis. Intracellular staining was performed on PBMNC (peripheral blood mononuclear cells), obtained following density gradient centrifugation of fresh blood samples. In details, PBMNC were fixed in formaldehyde 2%, and stained with fluorochrome-conjugated mAbs in presence of saponin 0,5%. For the evaluation of intracellular cytokines production, PBMNC were previously polyclonally stimulated for 5 hours with PMA and ionomycin, the last 3 in presence of brefeldin A[2]. Evaluation of Ki67 expression was performed following the manufacturer's instructions of anti-Human Foxp3 Staining Set (eBioscience) for fixing and staining details[3].

Determination of serum cytokines and chemokines concentration with bead-based multiplex immunoassays

Sera were collected and stored at -20°C. The quantitative determination of IL-1beta, IL-1Ralpha, IL-2, IL-4, IL-5, IL-6, IL-7 IL-8, IL-9, IL-10, IL-12, IL-13, IL-15, IL-17A, IFN-gamma, TNF-alpha, G-CSF, GM-CSF, VEGF, PDGF, FGF, IP-10, MCP-1, RANTES, eotaxin, MIP-1-alpha, and MIP-1-beta in the serum was performed by using a bead-based multiplex immunoassay (Biorad Laboratories) and the Bioplex 200 system (Biorad Laboratories) as previously described[2].

In brief, in a 96-well filter plate (Bio-Rad) 50µl of each serum sample were added to 50 µl of antibody-conjugated beads directed against the cytokines listed above (Bio-Rad). After a 30-min incubation, the plate was washed and 25 µl of biotinylated anti-cytokine antibody solution were added to each well

before another 30-min incubation. The plate was then washed and 50 µl of streptavidin-conjugated phycoerithrin were added to each well. After a final wash, each well was resuspended with 125 µl of assay buffer (Bio-Rad) and analyzed by Bioplex 200 (Biorad Laboratories, Hercules, CA).

Standard curves were derived from various concentrations of the cytokine standards and followed the same protocol as the serum samples. The concentration of the 27 cytokines (pg/ml) in each serum sample was calculated using the Bioplex 200 software.

Table S1. List of fluorochrome mAbs used for flow cytometric analysis

Marker	Clone	Flurochrome	Supplier
CCR7	150503	Horizon™ V450	BDBioscience
CD16	3G8	FITC<br>PerCP-cy5.5	BDBioscience
CD19	SJ25C1	APC	BDBioscience
CD3	UCHT1	Pacific Blue™<br>Horizon™ V450	BDBioscience
CD3	SK7	PE	BDBioscience
CD4	SK3	PE-Cy™7<br>PerCP	BDBioscience
CD45	2D1	Horizon™ V500	BDBioscience
CD45RA	L48	FITC	BDBioscience
CD56	MY31	PE	BDBioscience
CD56	NCAM16.2	PE	BDBioscience
CD56	B159	APC	BDBioscience
CD8	SK1	APC-Cy™7<br>APC<br>APC-H7	BDBioscience
HLA-DR	L243	FITC<br>Horizon™ V450	BDBioscience
IFN-γ	45-15	APC	Miltenyi Biotech
IFN-γ	25723.11	FITC	BDBioscience
TNF-α	6401.1111	FITC	BDBioscience
Ki67	20Raj1	PE	Thermo Fisher Scientific
CD15	MMAC	FITC	BDBioscience
CD123	9F5	PE	BDBioscience
CD64	10,1	PerCP-cy5.5	BDBioscience
CD13	L138	PE-Cy™7	BDBioscience
CD11b	D12	APC	BDBioscience
CD14	MϕP9	FITC	BDBioscience
CD33	P676	PE-Cy™7	BDBioscience
CD300	UP-H2	APC	BDBioscience
CD1c	AD5-8E7	PE	Miltenyi Biotech
CD141	1A4	APC	BDBioscience
CD66b	G10F5	Horizon™ V450	BDBioscience

Table S2. Baseline predictors of clinical improvement

Variable	Hazard Ratio (95% CI)
**Age, per years**	1.0 (0.9-1.0)
<60 years	-
60 to <70 years	3.1 (0.6-14.9)
70 to <80 years	1.1 (0.3-4.3)
≥ 80 years	1.5 (0.4-5.4)
**Female sex**	0.8 (0.4-1.7)
**Duration of symptoms prior to ruxolitinib treatment**	0.9 (0.9-1.0)
**Comorbidities**	
Neoplasia	0.5 (0.2-1.2)
Hypertension	0.5 (0.2-1.2)
Diabetes mellitus	0.4 (0.2-1.3)
Cardio vascular diseases	1.0 (0.5-2.1)
Pulmonary diseases	1.2 (0.5-2.7)
Neurological diseases	0.8 (0.4-1.6)
Autoimmune diseases	1.6 (0.5-4.8)
**Laboratory tests**	
Lymphocytes, per x10^9/L	0.9 (0.4-2.4)
C-reactive protein, per mg/l	1.0 (0.9-1.0)
D-Dimer, per ng/ml	1.0 (0.9-1.0)
Ferritin, per mg/l	1.0 (0.9-1.1)

CI, 95% Confidence Interval

Table S3: Longitudinal evaluation of leukocyte subsets in COVID-19 patients during treatment with ruxolitinib.

Cell Population	Controls	T0 (cells/µl)	T7 (cells/µl)	T14 (cells/µl)
Neutrophils	3.79 (±1.17)	4,96 (±2.75)	4.18 (±2.55)	4.29 (±2.38)
Lymphocytes	1.83 (±0.59) ***	0.96 (±0.45)	1.05 (±0.53)	1.25 (±0.62) *
Monocytes	0.53 (±0.10) *	0.37 (±0.20)	0.36 (±0.18)	0.46 (±0.16) *
Eosinophils	0.19 (±0.09) ***	0.03 (±0.07)	0.07 (±0.08) *	0.08 (±0.1) *
Basophils	0.03 (±0.02)	0.02 (±0.03)	0.03 (±0.07)	0.03 (±0.01)
Myeloid DC	0.0133 (±0.0045) ***	0.0026 (±0.0018)	0.0049 (±0.0032) *	0.0093 (±0.0042) ***
Plasmacytoid DC	0.0106 (±0.0059)	0.0064 (±0.01)	0.0043 (±0.0038)	0.0110 (±0.011) **

* p<0.05 versus T0

** p<0.01 versus T0

*** p<0.001 versus T0

Table S4: Longitudinal evaluation of serum cytokine levels in COVID-19 patients during treatment with ruxolitinib.

Cytokine	Controls (pg/ml)	T0 (pg/ml)	T7 (pg/ml)	T14 (pg/ml)
**IL-1β**	0.088 (±0.022) *	0.57 (±0.39)6.5	0.63 (±0.31)	0.139 (±0.038) ***
**IL-1rα**	24,6 (±17.1)	2117.37 (±4015.37)86	2024.35 (±4441.62)	288.47 (±537.79)
**IL-2**	0.14 (±0.17) **	3.70 (±1.97)9.3	3.27 (±1.91)	6.58 (±23.84)
**IL-4**	0.40 (±0.37) **	3.52 (±2.04)8.8	3.22 (±1.81)	0.87 (±0.51) ***
**IL-5**	Undetected	5.66 (±7.80)	3.27 (±5.43)	0.78 (±1.32) *
**IL-6**	0.05 (±0.05) *	4.48 (±3.28)89.6	2.1 (±1.49) *	0.48 (±0.55) ***
**IL-7**	Undetected	Undetected	Undetected	Undetected
**IL-8**	1.88 (±1.46) *	25.95 (±17.53)13.7	32.92 (±31.78)	7.06 (±6.75) ***
**IL-9**	0.32 (±0.41)	4.12 (±6.45)13.8	3.45 (±3.66) *	0.71 (±0.75) **
**IL-10**	1.18 (±0.13) ***	8.47 (±2.70)7.2	5.45 (±1.47) ***	1.27 (±0.25) ***
**IL-12**	0.51 (±0) ***	3.50 (±1.14)7	3.09 (±0.91)	0.78 (±0.27) ***
**IL-13**	0.227 (±0.277) *	2.56 (±1.73)11.6	2.02 (±1.31)	0.32 (±1.24) ***
**IL-15**	Undetected	Undetected	Undetected	Undetected
**IL-17A**	1.35 (±0.53) *	10.31 (±7.10)7.6	9.41 (±4.10)	2.44 (±0.98) ***
**Eotaxin**	19.09 (±18.11) *	113.52 (±73.59)5.9	124.68 (±84.67)	39.42 (±27.02) ***
**FGF**	8.52 (±2.76) ***	45.78 (±8.62)5.5	43.10 (±9.88)	13.08 (±5.00) ***
**G-CSF**	Undetected	Undetected	Undetected	Undetected
**GM-CSF**	Undetected	Undetected	Undetected	Undetected
**IFN-γ**	Undetected	2.13 (±3.64)	2.09 (±5.23)	0.10 (±0.25) *
**IP10**	53.61 (±57.53) **	4689.77 (±3134.34)87.5	1938.24 (±2044.08) **	153.37 (±91.25) ***
**MCP1**	13.48 (±10.41)	163.78 (±190.66)54.3	119.50 (±178.12)	18.38 (±19.36) **
**MIP1A**	0.41 (±0.41) **	5.12 (±2.95)12	7.56 (±4.50)	1.99 (±1.11) **
**MIP1B**	28.14 (±9.78) ***	114.44 (±21.15)4.1	124.09 (±28.59)	34.35 (±4.05) ***
**PDGF**	1125.53 (±808.97)	2481.46 (±1648.44)2.2	3110.84 (±2268.37)	832.84 (±495.67) ***
**Rantes**	2981.05 (±2146.47)	12729.1 (±17247.1)4.3	12910.8 (±7519.04)	3747.64 (±1726.37) *
**TNF-α**	4.92 (±1.12) **	39.03 (±20.82)9.5	33.87 (±13.07)	8.09 (±1.98) ***
**VEGF**	Undetected	Undetected	Undetected	Undetected

* p<0.05 versus T0; ** p<0.01 versus T0; *** p<0.001 versus T0

Figure S1. Patient Disposition.

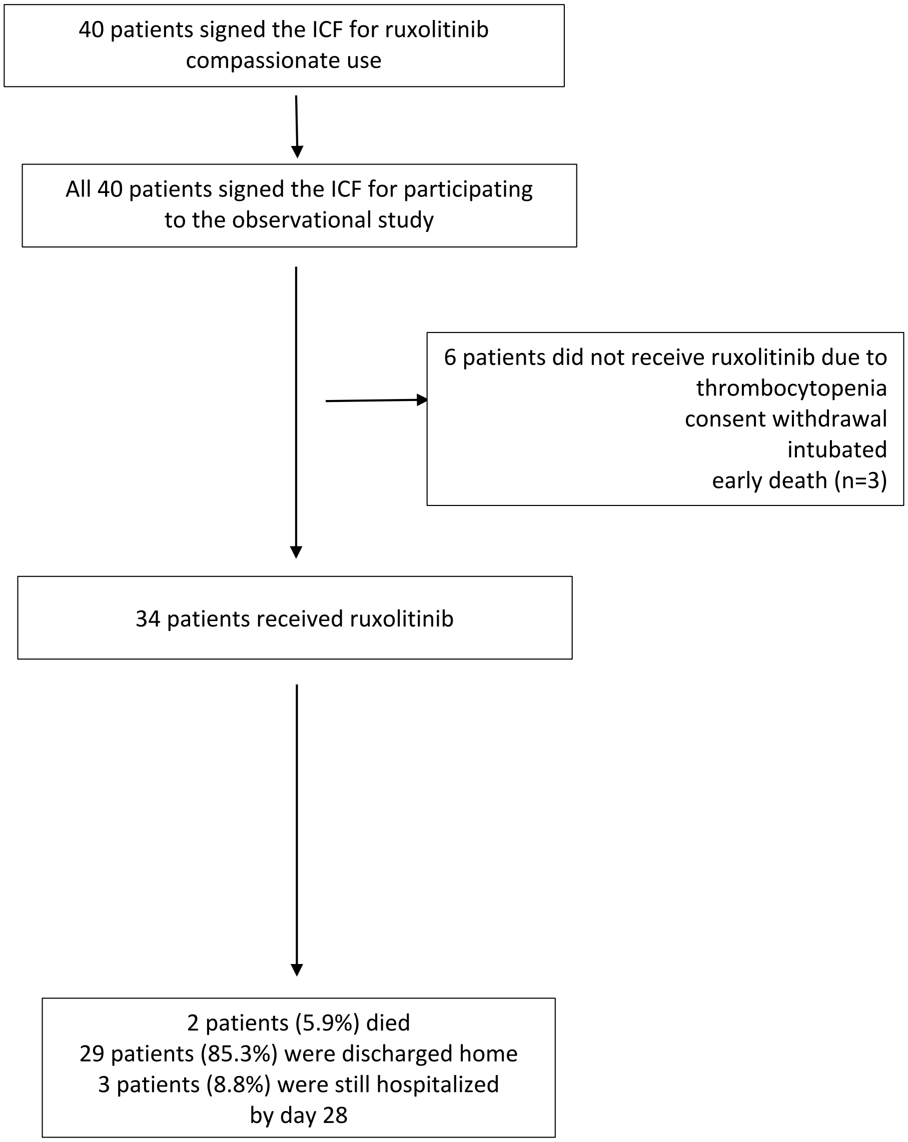

ICF= Informed Consent Form

References

1. Cossarizza A, Chang HD, Radbruch A, et al. Guidelines for the use of flow cytometry and cell sorting in immunological studies (second edition). Eur J Immunol 2019;49:1457-973.

2. Ledee N, Lombroso R, Lombardelli L, et al. Cytokines and chemokines in follicular fluids and potential of the corresponding embryo: the role of granulocyte colony-stimulating factor. Hum Reprod 2008;23:2001-9.

3. Soares A, Govender L, Hughes J, et al. Novel application of Ki67 to quantify antigen-specific in vitro lymphoproliferation. J Immunol Methods 2010;362:43-50.
